# The intelligent imaging revolution: artificial intelligence in MRI and MRS acquisition and reconstruction

**DOI:** 10.1007/s10334-024-01179-2

**Published:** 2024-06-20

**Authors:** Thomas Küstner, Chen Qin, Changyu Sun, Lipeng Ning, Cian M. Scannell

**Affiliations:** 1 Medical Image and Data Analysis (MIDAS.Lab), Diagnostic and Interventional Radiology, University Hospital of Tuebingen, 72076 Tuebingen, Germany; 2 Department of Electrical and Electronic Engineering, I-X Imperial College London, London, UK; 3 Department of Chemical and Biomedical Engineering, Department of Radiology, University of Missouri-Columbia, 65201 Columbia, USA; 4 Brigham and Women‘ s Hospital, 02215 Boston, USA; 5 Biomedical Engineering, Eindhoven University of Technology, Eindhoven, Netherlands

## Background

In the field of medical imaging, machine learning and artificial intelligence (AI) have emerged as techniques that are likely to fundamentally transform clinical practice in the coming years [1–7]. In the context of magnetic resonance imaging (MRI) and magnetic resonance spectroscopy (MRS), AI has the potential to impact all stages of the imaging pipeline, from (1) image acquisition and reconstruction to (2) image analysis and interpretation and (3) diagnosis and prognosis. If realized, this intelligent imaging revolution will lead to accelerated acquisition times, reduced workload for clinicians, reduced costs to the healthcare system, and more personalized treatment decisions for patients.

Due to the increasing availability of data and computing power, numerous AI solutions, primarily based on deep learning, have been proposed over the past few years, and methods are now evolving toward clinical application and usage. AI algorithms have been proposed and studied in the context of scan planning, accelerated acquisition and reconstruction, and image analysis. While current work often focuses only on one part of this full imaging pipeline, deep learning provides many more opportunities to improve the whole workflow of MR image from acquisition to analysis and diagnosis. Future investigations of deep learning approaches will further support the choice of the examination based on actual physiological scan parameters, e.g., heart rate, or on the patient information obtained during the planning. Furthermore, deep learning techniques will support further acceleration in scan time, to allow for real-time interventional MRI [8]. We observe a trend toward embedding different elements of the imaging pipeline (acquisition, reconstruction, post-processing, analysis, diagnosis) in deep learning models and training a network end to end in so-called multi-task networks, or to exploit the additionally available data, and these directions will form the future of learning-based MR imaging. However, challenges relating to the usability, robustness, and reliability of AI algorithms are crucial when used in daily clinical practice, and their performance needs to be ensured and validated for diverse and heterogeneous patient cohorts.

This special issue highlights current developments and unresolved challenges in the push to move AI from a hot research topic to a clinical reality for MRI/MRS acquisition and reconstruction.

## Acquisition and reconstruction

MR imaging represents a significant opportunity for AI due to the redundancy and the high dimensionality of the data. AI may help to overcome challenges regarding acquisition time, SNR, the trade-off between spatial and temporal resolutions, and different types of artifacts, e.g., cardiac, and respiratory motion. While deep learning approaches often outperform conventional approaches in terms of pixel-wise quantitative metrics, these approaches can be overconfident or overfit to the underlying application, and therefore attention needs to be paid to the real-world evaluation of these models to ensure reliability for clinical deployment. It is challenging to evaluate the quality and robustness of AI reconstruction approaches, especially for subtle pathologies. In particular, we may find overly optimistic results by using simulated data and neglecting the unprocessed raw k-space data [9].

The robustness of neural networks to changes in anatomy [10] and architectures [11] was studied in the context of static 2D imaging. Small adversarial input perturbations can affect the neural networks differently [12]. Domain shifts in anatomy have also been shown to impact image reconstruction when using unrolled networks and moderate acceleration [10], but are more subtle and difficult to identify. This observation regarding domain shift is different to image analysis tasks, where a subtle change in data characteristics might already lead to, e.g., mis-segmentation. However, as reconstructions serve as the basis for further downstream tasks and considering that when deployed in a clinical environment these solutions might be more likely to encounter out-of-domain data, e.g., other imaging sequences or contrasts, or patients with different pathologies, biases and error propagation could occur. Clearly, hurdles remain to be overcome and better quality assurance mechanisms need to be developed.

In this issue, Heckel et al. provide a very comprehensive review of recent advances in AI reconstruction for robust MRI [13]. They particularly focus on recent advancements in the field where approaches such as end-to-end network training, generative priors, and self-supervised learning play key roles. They discuss new neural network architectures, such as transformers, that are sure to play an increasing role in the field in the coming years. They continue to consider the possibility of AI for image acquisition to be developed in tandem with AI reconstruction algorithms to improve k-space sampling and pulse sequence design. In addition, they introduce advanced applications, such as quantitative and dynamic MRI, and consider the main challenges of AI-based reconstruction which include hallucinations, model instabilities, the difficulty of benchmarking, how to quantify uncertainty, and the need for large diverse dataset for both model development and validation.

Villegas-Martinez et al. and Yang et al. provide focused reviews on cardiovascular [14] and neurological [15] applications, respectively. With respect to the acquisition, common themes which persist regardless of the application are automated scan planning, acquisition parameter selection, and artifact correction, with methods to improve workflow efficiency and reduce operator variability promising to lead to a future with faster, lower-dose and contrast agent-free imaging. Zhou et al. specifically address one of the main challenges of MR acquisition, subject motion, and discuss the use of AI to correct for this [16]. They survey AI methods to reduce or estimate motion artifacts in both image and frequency domains. In addition, they consider how the estimated motion can be used and how possible synergies between the motion estimation and the downstream tasks (e.g., segmentation, parametric mapping, or MR-guided therapy) can be exploited. In keeping with the trend of the other work in this issue, they identify the pressing need to be able to properly validate the accuracy and fidelity of the estimated motion.

Some of the previously discussed challenges are also tackled within this special issue. Original research from Venkatesh et al. introduces SpiNet-QSM for reconstructing quantitative susceptibility maps (QSM) using a Schatten p-norm model-based AI framework [17]. Of particular interest is the learnable *p* parameter which allows the framework the flexibility to adapt the regularizer to the specific data.

Two studies address hardware limitations with (1) Zhang et al. proposing a physics-guided AI model in 2.5D that yields high-quality coronary MRI reconstructions based on quantitative image quality metrics and the assessment of expert cardiologists as compared to conventional 3D kooshball coronary MRI [18], and (2) Shafique et al. presenting a parallel framework for SVD-based low-rank model for reconstruction of highly accelerated dynamic cardiac MR that achieves > 10 × speedup in computation time [19].

The work of Fujima et al. investigates the obtained image quality and quantitative reliability of model-based deep learning reconstructions in diffusion-weighted imaging. They found significant qualitative and quantitative, in terms of SNR and CNR, differences between conventional and AI-based reconstructions [20].

In the domain of MRS reconstruction, Berto et al. report the results of a conference challenge that aimed to achieve faster imaging time with the use of AI reconstruction [21]. Specifically, the reconstruction focused on gamma-aminobutyric acid (GABA)-edited MRS with just one-quarter of the typical transients. A range of different neural network architectures and training schemes achieved promising results, but the authors acknowledge the challenge of assessing the quality of spectral reconstructions.

## Image analysis and interpretation

Post-processing is the most widely studied application of AI in medical imaging. In particular, image analysis tasks such as segmentation and landmark detection are now routinely tackled with AI. It is probable that post-processing AI applications are more mature as they are easier to evaluate compared to acquisition/reconstruction tasks and there tends to be more data available to train such models. For example, quantitative metrics of evaluating automated segmentations are well established, whereas we have seen that evaluating reconstructed images remains a challenge [22]. However, questions about the reliability of AI are still present for post-processing applications in the presence of biases and domain shifts [23].

In line with previously discussed trends, image analysis tasks are now being tackled with joint models using multi-task learning, linking the analysis with the acquisition and reconstruction of the images. Prominent examples of this are the combined reconstruction and segmentation of accelerated MRI, which have been proposed for several different sequences and anatomies [24–28]. Similar synergies between acquisition and image analysis are being made for other analysis steps as well, such as kinetic parameter estimation where research is studying the direct reconstruction of parametric maps from k-space, linking the acquisition and reconstruction directly to the diagnostic task [3, 29]. We envisage this trend to continue and expand in the future, potentially leading to all reconstruction and image analysis tasks being completed by one model in an end-to-end fashion.

Novel learning strategies [30] and the increasing availability of data also foster the trend toward more complex architectures. These networks can better generalize to different domains [31] and/or serve a multitude of tasks [32]. Although this generalizability aspect is desirable, clinical reliability and practical value still need to be investigated.

In this special issue, Suwannasak et al. make such a step to evaluate an AI-based super-resolution algorithm. In particular, the algorithm allows generation of high-resolution images from 2× under sampled low-resolution 3D T1-weighted brain MR images. The authors show the potential to reduce scan time to just 1 min, while their analysis shows that the super-resolution images still give accurate brain volume measurements and preserve image quality [33].

Other analysis applications are presented by Huang et al. who present work on metabolite concentration quantification for in vivo MRS, and their promising results with a CNN-based model aligned well with the ground truth but required further investigation for differentiating metabolites with low concentrations from overlapping signals [34]. Kafali et al. quantified visceral and subcutaneous adipose tissue based on a 3D multi-contrast CNN and achieved excellent performance based on longitudinal data suitable for monitoring the risk factors for cardiometabolic diseases over time in overweight patients [35].

## Image diagnosis and prognosis

The advancement of AI has also opened new frontiers to potentially improve the diagnostic accuracy and prognostic capabilities. Developments are crucial for accurate disease characterization and early treatment planning across various specialties, such as oncology, neurology, and cardiovascular imaging. Radiomics and ML models have been leveraged to extract image features [36–38], which provide insights into disease characteristics that may be neglected through conventional methods. Additionally, the use of multiparametric MRI protocols facilitates detailed tissue characterization, aiding in the early disease detection [39–41], risk stratification [42, 43], or therapy monitoring and guidance [44, 45]. To increase the model robustness and reliability, techniques of data augmentations and synthetic MR images generation were developed [46, 47] to address the need for large and diverse datasets for training. Moreover, hybrid AI models that integrate deep learning with radiomics offer enhanced diagnostic precision and more detailed prognostic assessments from MRI scans [48].

However, operating in a diagnostic and prognostic setting poses several challenges. The diversity of MRI data across different machines and protocols complicates the development of universally applicable AI models. AI applications in the clinical environment require high reliability and reproducibility, which necessitates clinical validations, standardized datasets, and comparisons across MRI machines and sites. The integration of these models into existing clinical workflows requires not only technical compatibility—and in some cases major infrastructural changes—but also substantial training for clinicians to ensure accurate and effective use of these AI tools.

The risk of misdiagnosis emphasizes the need for clinicians to apply and triage their judgment with AI recommendations. Human-interpretable feedback about the output and/or processing of the data needs to be provided and analyzed, to increase understanding and collaboration. Another risk relates to training on imbalanced datasets which can lead to biases in diagnosis and prognosis such as inaccurate predictions for underrepresented patient groups. Furthermore, issues surrounding privacy and security cannot be neglected, given the sensitive nature of patient data involved in these AI systems. It is crucial that we ensure the benefits of AI in MRI without compromising ethical standards and patient care quality.

While the potential benefits of diagnostic and prognostic models are large, so too are these risks and challenges, and as such, these use-cases of AI are proportionally underrepresented for MRI/MRS data. They are also not included in this special issue, but the universal theme of the need robust validation of AI in these cases remains clear.

This special issue does, however, provide valuable insights into a potentially crucial step in this process, the model evaluation. In the paper by Sharma et al., different statistical tests were used for the comparison of deep learning models trained with different hyperparameter settings and it was found that mixed effect models may be more sensitive than ANOVA for identifying such differences [49].

In summary, the papers of this special issue cover a spectrum of applications from the acquisition to the analysis of MRI/MRS data. Together, they illustrate the power of AI and identify the steps that still need to be taken for the full potential impact of AI to be felt in routine clinical practice. As discussed, many of these wheels are already in motion and given the pace of current progress, and sooner rather than later AI algorithms for MRI/MRS acquisition and reconstruction will be in widespread use in hospitals, benefiting patients and clinicians.
